# The Efficiency of Direct Maturation: the Comparison of Two hiPSC Differentiation Approaches into Motor Neurons

**DOI:** 10.1155/2022/1320950

**Published:** 2022-12-09

**Authors:** Catherine Schaefers, Simone Rothmiller, Horst Thiermann, Theo Rein, Annette Schmidt

**Affiliations:** ^1^Bundeswehr Institute of Pharmacology and Toxicology, Neuherbergstr. 11, 80937 Munich, Germany; ^2^Max Planck Institute of Psychiatry, Kraepelinstr. 2-10, 80804 Munich, Germany; ^3^Institute of Sport Science, University of the Bundeswehr Munich, Werner-Heisenberg-Weg 39, 85577 Neubiberg, Germany

## Abstract

Motor neurons (MNs) derived from human-induced pluripotent stem cells (hiPSC) hold great potential for the treatment of various motor neurodegenerative diseases as transplantations with a low-risk of rejection are made possible. There are many hiPSC differentiation protocols that pursue to imitate the multistep process of motor neurogenesis *in vivo*. However, these often apply viral vectors, feeder cells, or antibiotics to generate hiPSC and MNs, limiting their translational potential. In this study, a virus-, feeder-, and antibiotic-free method was used for reprogramming hiPSC, which were maintained in culture medium produced under clinical good manufacturing practice. Differentiation into MNs was performed with standardized, chemically defined, and antibiotic-free culture media. The identity of hiPSC, neuronal progenitors, and mature MNs was continuously verified by the detection of specific markers at the genetic and protein level via qRT-PCR, flow cytometry, Western Blot, and immunofluorescence. MNX1- and ChAT-positive motoneuronal progenitor cells were formed after neural induction via dual-SMAD inhibition and expansion. For maturation, an approach aiming to directly mature these progenitors was compared to an approach that included an additional differentiation step for further specification. Although both approaches generated mature MNs expressing characteristic postmitotic markers, the direct maturation approach appeared to be more efficient. These results provide new insights into the suitability of two standardized differentiation approaches for generating mature MNs, which might pave the way for future clinical applications.

## 1. Introduction

Human-induced pluripotent stem cells (hiPSC) are of great importance for regenerative medicine, as a wide variety of cell types can be generated, avoiding the controversially discussed use of embryonic stem cells (ESC). Transplantations with a low-risk of immunological rejection are made possible with autologous hiPSC-derived cells. Furthermore, reprogramming of cells from diseased individuals to hiPSC allows the analysis of pathomechanisms, e.g., of Alzheimer's disease, to be investigated and sets the course for specific drug discovery. With patient-derived hiPSC, it may soon be possible to predict the patient's individual response to drug-specific therapy [1].

Currently, there is no appropriate therapy available for amyotrophic lateral sclerosis (ALS), a neuromuscular disease in which degeneration of motor neurons (MNs) occurs. Replacement of those degraded MNs would be desirable for the treatment of ALS but also other diseases involving degenerated, dysfunctional, or missing MNs. hiPSC are a promising tool for motor neuron (MN) generation, which exhibit the same MN differential potential in comparison to ESC. For clinical application, the generation of human mature and functional MNs needs to underly ensures quality and reproducibility with minimum use of xenogeneic material [2]. Thus, the selection of hiPSC induction method is a critical factor. Existing MN differentiation protocols use, e.g., genomic-integrating lenti- [3] or retroviruses [4, 5] for hiPSC reprogramming which is questionable because of their mutagenic potential [6]. Other protocols are performed with, e.g., nongenomic-integrating sendai virus [7] but apply feeder-cells or antibiotics for maintaining hiPSC, having a not yet fully investigated impact on hiPSC differentiation potential [8]. In addition, the selection of reprogramming factors and type of cells used for hiPSC reprogramming is decisive. Reprogramming factors like C-myc, being described as oncogenic, need to be replaced by factors with comparable reprogramming efficiency such as L-myc [9]. The epigenetic memory of reprogrammed cells also has to be considered as it might affect the differentiation potential of generated hiPSC [10]. The process of hiPSC differentiation into MNs still seems to be challenging as there are no defined differentiation media in certified, ready to use-quality available, and postmitotic, xeno-free hiPSC-derived MNs were not purchasable at the beginning of this study.

Various hiPSC differentiation protocols aim to mimic motor neurogenesis *in vitro*. Bianchi et al. [11], e.g., pursue a three-step differentiation strategy to generate functional MNs, starting with neural induction via inhibition of BMP and TGF*β*/Activin/Nodal pathways (dual-SMAD inhibition) followed by MN differentiation via, i.e., retinoic acid (RA) and sonic hedgehog (SHH) exposure, and subsequent MN maturation. The use of feeder cells limits this differentiation protocol in its implementation in regenerative medicine. A similar three-step sequence is used in the protocols of Stemcell Technologies (Vancouver, Canada) applying standardized, defined, and antibiotic-free culture media for neural induction, differentiation, and maturation. Interestingly, previous studies demonstrated that neurons with caudal positional identity can be generated by inhibition of TGF*β*/Activin/Nodal-signalling, which acquire electrophysiological competence in the further course of cultivation [12, 13]. This might be achieved by STEMDiff™ SMADi Neural Induction Kit and subsequent maturation.

This study is aimed at comparing this two-step direct maturation approach, named approach A, with the three-step protocol, named approach B, in terms of its efficiency to generate mature MNs from hiPSC. To approach the requirements of clinical application, an adapted reprogramming cocktail and virus-, feeder-, and footprint-free method were used for hiPSC generation. Serum- and antibiotic-free culture medium manufactured under clinical good manufacturing practice (cGMP) was applied for maintaining hiPSC. In the further course of differentiation, chemically defined and standardized culture media were used to ensure a robust and reproducible protocol. Differences on morphological, genetic, and protein levels were investigated, which might provide new insights into a more efficient differentiation method that approaches applications in regenerative medicine.

## 2. Materials and Methods

### 2.1. Generation of hiPSC via Nucleotransfection of Human Neonatal Dermal Fibroblasts

Human neonatal dermal fibroblasts (hPDF) (ATCC, Manassas, Virginia, USA) were cultured in Fibroblast Growth Kit-Low serum (FGM) (ATCC, Manassas, Virginia, USA) until maximum passage 3 according to manufacturer's protocol. On day 0, episomal nucleotransfection with three plasmids encoding reprogramming factors and one plasmid encoding Green Fluorescent Protein (GFP) (see Supplementary Method 1 and 2) as control (Addgene, Watertown, Massachusetts, USA) was performed with Amaxa® Nucleofactor II Device (Lonza, Basel, Switzerland) due to manufacturer's instructions. Briefly, 1 × 10^6^ hPDFs were transfected with 1 *μ*g DNA of each of the four plasmids (total 4 *μ*g DNA). As a transfection control, 2 *μ*g of pmaxGFP®-Vector (Lonza, Basel, Switzerland) was introduced into the cells according to the manufacturer's directions. After changing the media on day 1, transfected cells were captured by ZEISS LSM 710 confocal microscope (ZEISS, Oberkochen, Germany). On day 2, cells were passaged by washing once with PBS, adding Gibco® Trypsin 0.05%-EDTA (Thermo Fisher, Waltham, Massachusetts, USA) and incubating for 5 min at 37°C in a humidified atmosphere containing 5% CO_2_. Trypsination was stopped by adding medium. Cells were centrifuged for 5 min at 526 × g and plated 1 : 6. On day 4, cells were passaged as described before and plated on Corning® Matrigel® precoated plates. For maintaining hiPSC, cells were cultivated for 30 days in mTeSR™1 (produced under cGMP by Stemcell Technologies, Vancouver, Canada) with a medium change every other day. Passaging was performed with Gentle Cell Dissociation Reagent (GCDR, produced under cGMP by Stemcell Technologies, Vancouver, Canada) every 5 to 7 days. In brief, cells were washed once with PBS and incubated for 10 min with GCDR. Supernatant was removed and mTeSR™1 supplemented with 10 *μ*M Y-27632 (Stemcell Technologies, Vancouver, Canada) was added. hiPSC were collected by rinsing in a circular pattern and were plated 1 : 4 on Corning® Matrigel® precoated plates. The identity of hiPSC was confirmed by alkaline phosphatase live staining, immunofluorescence, and gene expression analysis (see Supplementary Method 3-6).

### 2.2. Differentiation of hiPSC into Neural Progenitor Cells (NPC)

For neuronal induction, hiPSC were treated with STEMDiff™ SMADi Neural Induction Kit (Stem Technologies, Vancouver, Canada) for 21 days with a medium change every other day and passaged with STEMDiff™ Neural Rosette Selection Reagent (Stemcell Technologies, Vancouver, Canada) every 5 to 7 days onto precoated poly-L-ornithine/laminin plates (both Sigma-Aldrich, St. Louis, Missouri, USA). The expansion phase of neural progenitor cells was initiated with STEMDiff™ Neural Progenitor Medium (NPM, Stemcell Technologies, Vancouver, Canada) with a medium change every other day. StemPro™ Accutase™ Cell Dissociation Reagent (Thermo Fisher, Waltham, Massachusetts, USA) was used for passaging cells by incubating for 5 min at 37°C in a humidified atmosphere containing 5% CO_2_ and centrifuging for 5 min at 296 × g. Cells were maintained in NPM for 21 days. 3 × 10^6^ derived NPC were cryopreserved in 1 mL STEMDiff™ Neural Progenitor Freezing Medium (Stemcell Technologies, Vancouver, Canada) and stored at -150°C.

### 2.3. Two Different Procedures to Differentiate NPC

For the approach A, NPC were cultured in STEMDiff™ Neuron Maturation Medium (Stemcell Technologies, Vancouver, Canada) for 28 days with a change of medium every other day.

Approach B was first treated with STEMDiff™ Neuron Differentiation Medium for 7 days with a daily medium change. Cells were then exposed to STEMDiff™ Neuron Maturation Medium (Stemcell Technologies, Vancouver, Canada) for 21 days with a change of medium every other day.

### 2.4. Differences in Neuronal Clustering Captured by the IncuCyte®

500,000 cells of approach A and B were plated per well on poly-L-ornithine/laminin precoated 6-well plates. To observe the differences in the capability of forming neuronal networks, the plates were placed in the IncuCyte® S3 system (Sartorius, Göttingen, Germany). Cells were imaged two times a day over a period of 28 days. The experiment was performed in triplicate.

### 2.5. Test for Expression of Neurofibrils

For staining of neurofibrils with Bielschowsky (Bio-Optica, Milan, Italy), NPC and cells differentiated over approaches A and B for 28 days were seeded onto poly-L-ornithine/laminin (Sigma-Aldrich, St. Louis, Missouri, USA) precoated Nunc™ Lab-Tek™ II Chamber Slides™ (Thermo Fisher, Waltham, Massachusetts, USA), fixed with 4% w/v PFA in PBS by incubation for 20 min at 4°C and washed twice with PBS. According to the manufacturer's protocol, the slide was washed twice with ultrapure water and was incubated with 10 drops of Reagent A for 15 min at 40°C. After washing two times with ultrapure water, 10 drops of Reagent B were added following incubation for 20 min at 50°C. The supernatant was discarded, and the slide was treated with reduction solution (20 drops Reagent C, 8 drops each of Reagents D, E, and F in 50 mL ultrapure water) for 2 min. Cells were washed twice with ultrapure water and subsequently incubated with 10 drops of Reagent G for 3 min. Before dehydrating the slide with ascending concentration of ethanol and treating twice with Xylene (Sigma-Aldrich, St. Louis, Missouri, USA), it was washed two times with ultrapure water. Finally, the slide was embedded in Entellan Neu (Merck, Darmstadt, Germany) following incubation for 30 min at room temperature to dry. The stained neuronal structures were imaged with Nikon Eclipse TS100 microscope (Nikon, Minato, Japan).

### 2.6. Flow Cytometric Analysis with Neuronal Markers to Determine Differentiation Status

For fluorogenic staining, 250,000 NPC and cells of approaches A and B differentiated for 28 days were fixed by carefully resuspending in 300 *μ*L of 4% w/v PFA in PBS and incubating for 20 min at 4°C. Subsequently, 1 mL perm solution (0,1% v/v Triton X-100 and 0,3% w/v sodium dihydrogen citrate ((Sigma-Aldrich, St. Louis, Missouri, USA) in ultrapure water) was added and cells were centrifugated for 5 min at 296 × g. The supernatant was discarded and permeabilization was performed by careful resuspension in 300 *μ*L perm solution and incubation for 2 min at 4°C. Cells were pelleted (5 min at 296 × g), resuspended in 45 *μ*L FACS solution (PBS with 2% v/v FBS), and subsequently stained with the following labelled antibodies by incubation for 30 min in the absence of light: Nestin PerCP-Cy™5.5 (5 *μ*L, Becton Dickinson, Franklin Lakes, USA), ChAT-APC (5 *μ*L of 1 : 500 in FACS solution, abcam, Cambrigde, UK), and DAPI (5 *μ*L of 1 : 1,000 in FACS solution, Biolegend, San Diego, California, USA). Unstained cells and stained cells with the following antibodies for fluorescent isotypes were taken along as controls: PerCP-Cy™5.5 (5 *μ*L, Becton Dickinson, Franklin Lakes, USA), IgG APC (5 *μ*L of 1 : 500 in FACS solution, abcam, Cambrigde, UK). After washing three times with 300 *μ*L FACS solution, 10,000 cells were analyzed using the Amnis® ImageStream®^X^ Mk II instrument with ISX Software (Luminex Corporation, Austin, USA). The experiment was performed three times independently and the data was processed and presented with the IDEAS® Software (Luminex Corporation, Austin, USA).

### 2.7. Immunofluorescence

For fixation, cells cultured on poly-L-ornithine/laminin precoated Nunc™ Lab-Tek™ II Chamber Slides™ were washed twice with PBS and were incubated with 4% w/v PFA in PBS for 20 min at 4°C. Slides were washed three times with Wash Buffer (Biozol, Eching, Germany) for 2 min with gentle shaking. Permeabilization was conducted by incubation with perm solution for 5 min at 4°C. After three times of washing for 2 min, cells were blocked for 30 min using serum-free Protein Block (Agilent, Santa Clara, California, USA). Following primary antibodies were diluted in background reducing Antibody Diluent (Agilent, Santa Clara, California, USA): Rabbit Anti-Peripherin (ab4666 abcam, Cambridge, UK) 1 : 1,000, Rabbit Anti-NF-H (ab8135, abcam, Cambridge, UK) 1 : 2,000, Rabbit Anti-Synapsin 1 (LS-C117933 LifeSpan Biosciences, Seattle, Washington, USA) 1 : 500, Rabbit Anti-Pax6 (HPA030775 Atlas Antibodies, Stockholm, Sweden) 1 : 500, Rabbit Anti-ChAT (ab223346 abcam, Cambridge, UK) 1 : 100, Rabbit Anti-MNX1 (LS-C148679 LifeSpan Biosciences, Seattle, Washington, USA) 1 : 200, recombinant Rabbit Anti-Islet 1 (ab109517 abcam, Cambridge, UK) 1 : 250, Mouse Anti-beta Tubulin 3 (GTX27751 Genetex, Irvine, California, USA) 1 : 1,000, Rabbit Anti-Nestin (HPA007007 Atlas Antibodies, Stockholm, Sweden) 1 : 1,000, and Mouse Anti-NeuN (GTX30773 Genetex, Irvine, California, USA) 1 : 100. Cells were incubated with diluted primary antibodies overnight at 4°C with gentle shaking. After washing three times for 2 min, the following secondary antibodies were added and incubated for 90 min with exclusion of light: Goat Anti-Rabbit IgG Antibody (H + L) Dylight® 488, Goat Anti-Mouse IgG Antibody (H + L) Dylight® 594 (Vector Laboratories, Burlingame, California, USA) both 1 : 800 and DAPI (422801, Biolegend, San Diego, California, USA) 1 : 75,000 as nucleic acid stain. The slides were washed four times for 2 min with ultrapure water, subsequently mounted in Prolong® Antifade (Invitrogen by Thermo Fisher Scientific, Waltham, Massachusetts, USA) and examined microscopically with a ZEISS LSM 710 confocal microscope (ZEISS, Oberkochen, Germany).

### 2.8. Western Blot

The method of Western Blot analysis used here is based on the protocol recently published by Horn et al. [14] and was carried out as follows. NPC and cells differentiated over approaches A and B for 28 days, cultured in 6-well plates, were washed once with PBS (Gibco, Thermo Fisher, Waltham, Massachusetts, USA), detached by incubation with StemPro™ Accutase™ Cell Dissociation Reagent (Thermo Fisher, Waltham, Massachusetts, USA) for 5 min at 37°C in humidified atmosphere containing 5% CO_2_, and centrifuged for 5 min at 296 × *g*. 2 × 10^6^ cells of each cell type were resuspended in 400 *μ*L of Tris-EDTA-Triton X-100 extraction buffer (6.25 mM TRIS, 12.5 mM NaCl, 2.5 mM EDTA, 1.5% Triton X-100, one Complete Mini Inhibitor Cocktail (Roche, Basel, Switzerland) and one PhosSTOP (Roche, Basel, Switzerland) in 10 mL ultrapure water) and were incubated on ice for 15 min. Cells were disrupted using an ultrasonic homogeniser (Bandelin Electronic, Berlin, Germany) by pulsing three times for 10 s, 0.3 interval, and 30% intensity, following incubation on ice for 1 h, with vortexing every 15 min. Cell debris was removed by centrifuging for 10 min at 17,186 × g at 4°C. Supernatant was transferred to a 0.5 mL Protein LoBind® Tube (Eppendorf AG, Hamburg, Germany) and kept on ice for determination of protein concentration using Qubit™ Protein Assay Kit with Qubit 4 Fluorometer (Thermo Fisher, Waltham, Massachusetts, USA) according to manufacturer's instructions. The cell lysate was aliquoted into units of 15 *μ*L and was stored at -20°C. Protein denaturation was conducted by mixing cell lysates containing 40 *μ*g protein, 8 *μ*L loading buffer (60% v/v of 4× Protein Sample Loading Buffer (LI-COR Biosciences, Lincoln, Nebraska, USA), and 3.12% w/v DTT ((Sigma-Aldrich, St. Louis, Missouri, USA) in ultrapure water) and ultrapure water to a total volume of 25 *μ*L per sample and subsequent incubation for 5 min at 95°C. For analysis of Pax6 and MNX1, samples and 3 *μ*L Chameleon™ Duo Prestained Protein Ladder (LI-COR Biosciences, Lincoln, Nebraska, USA) were loaded on NuPAGE™ 4-12% Bis–Tris gels 1.0 mm × 10 wells with NuPAGE™ MES SDS Running Buffer (both Novex by Thermo Fisher Scientific, Waltham, USA). For analysis of Nestin, samples and 7 *μ*L Spectra™ Multicolor High Range Protein Ladder (Thermo Fisher Scientific, Waltham, USA) were loaded on NuPAGE™ 3–8% Tris Acetate gels 1.0 mm × 10 wells with NuPAGE™ Tris-Acetate SDS Running Buffer (both Novex by Thermo Fisher Scientific, Waltham, USA). Gel electrophoresis was performed for 50 min at 200 V (Pax6, MNX1) or for 60 min at 150 V (Nestin) using a Mini Gel Tank (Invitrogen by Thermo Fisher Scientific, Waltham, USA).

Protein Transfer was conducted using iBlot™ Transfer Stack PVDF (0.2 *μ*m pore size) with iBlot™ 2 Gel Transfer Device (Thermo Fisher, Waltham, Massachusetts, USA) for Pax6 and MNX1 for 7 min (1 min 20 V, 4 min 23 V, and 2 min 25 V) and for Nestin for 10 min (2 min 20 V, 5 min 25 V, and 3 min 25 V). PVDF membranes were washed once in ultrapure water, reactivated by rinsing with methanol (Sigma-Aldrich, St. Louis, Missouri, USA) for 30 s and two times with ultrapure water, and subsequently blocked in Intercept® (PBS) Blocking Buffer (LI-COR Biosciences, Lincoln, Nebraska, USA) for 1 h with gentle shaking. Membranes were incubated overnight at 4°C with following primary antibodies diluted in antibody diluent (Intercept® (PBS) Blocking Buffer with 0.2% v/v Tween® 20 (Sigma-Aldrich, St. Louis, Missouri, USA)): Recombinant Rabbit Anti-MNX1 (ab79541, abcam, Cambridge, UK) 1 : 1,000, Rabbit Anti-Nestin (HPA007007, Atlas Antibodies, Stockholm, Sweden) 1 : 1,000, and purified Mouse Anti-Pax6 (MA1-109, Thermo Fisher, Waltham, Massachusetts, USA) 1 : 1,000. Subsequently, the membranes were washed two times for 10 min with washing buffer (PBS with 0.1% v/v Tween® 20). Secondary antibody incubation was performed for 90 min under light exclusion with IRDye® 800CW Goat Anti-Mouse or IRDye® 800CW Goat Anti-Rabbit (LI-COR Biosciences, Lincoln, Nebraska, USA) 1 : 8,000 in antibody diluent. Membranes were washed again two times for 10 min with washing buffer and rinsed two times with ultrapure water. For normalization with the house-keeping protein glyceraldehyde-3-phosphate dehydrogenase (GAPDH), the primary antibody recombinant Rabbit Anti-GAPDH (ab181602, abcam, Irvine, California, USA) 1 : 10,000 in antibody diluent was added and detected by secondary antibody Goat anti-Rabbit IRDye® 680RD (LI-COR Biosciences, Lincoln, Nebraska, USA) 1 : 8,000 in antibody diluent. The experiment was carried out three times independently.

For imaging the blots, the Odyssey CLx (LI-COR Biosciences, Lincoln, Nebraska, USA) imaging system was used. Densiometric analysis was performed with Empiria Studio® software version 2.2 (https://www.licor.com/bio/empiria-studio/resources). Analysis of each replicate sample was conducted after normalization of Western Blot data. Results were illustrated with the software RStudio (version 4.0.3 (2020-10-10), RStudio Inc., Boston, USA).

### 2.9. Analysis of Gene Expression

The method of gene expression analysis used here is based on the protocol described previously by Horn et al. [14] and was performed as follows. NPC and cells of approach A and B differentiated for 28 days were washed once with PBS and were obtained by using a cell scraper (Greiner AG, Kremsmünster, Austria) directly into RNA later (Qiagen, Hilden, Germany). RNA extraction was performed according to the manufacturer's instructions using RNeasy Mini Kit (Qiagen, Hilden, Germany). Briefly, RNA later was discarded, and cells were lysed under repeated resuspension with 600 *μ*L buffer RLT. The cell lysate was transferred to a QIAshredder spin column (Qiagen, Hilden, Germany) and centrifuged for 2 min at 21,135 × g. The eluate was mixed with 600 *μ*L of 70% ethanol. 700 *μ*L of this mixture was pipetted into a spin column and centrifuged for 30 s at 13,250 × g. The eluate was discarded, and the column was washed three times. Once with 700 *μ*L buffer RW1 (30 sec, 13,250 × g) and two times with 500 *μ*L buffer RPE (2 min, 21,135 × g). To dry the column, it was centrifuged at 21,135 × g for 1 min. RNA was eluted with 30 *μ*L nuclease free water (Qiagen, Hilden, Germany) by spinning 1 min at 21,135 × g in a Biopur® 1.5 mL tube (Eppendorf AG, Hamburg, Germany).

The NanoQuant Plate™ with Plate Reader infinite M200 Pro (Tecan Group AG, Männedorf, Switzerland) was used for RNA concentration determination. The amount of RNA for cDNA synthesis was set to 500 ng per qRT-PCR plate. cDNA synthesis was performed with the RT^2^ First Strand Kit (Qiagen, Hilden, Germany) according to the manufacturer's protocol. In brief, for elimination of genomic DNA, 2 *μ*L of buffer GE, RNA, and RNase free water were mixed to a total volume of 10 *μ*L and incubated for 5 min at 42°C followed by cooling on ice for at least 1 min. 10 *μ*L reverse transcription mix consisting of 4 *μ*L 5× buffer BC3, 1 *μ*L control P2, 2 *μ*L RE3 reverse transcriptase mix, and 3 *μ*L RNase free water was added to the genomic elimination mix. Reverse transcription was performed by incubation for 15 min at 42°C, followed by incubation for 5 min at 95°C to stop the process. The Mastercycler® nexus GX2 (Eppendorf AG, Hamburg, Germany) was used for all incubation steps. 91 *μ*L RNase free water was added to the preparation. The cDNA mix was stored at –20°C for a maximum of 7 days.

For the preparation of the detection mix and the pipetting of qPCR plates, the Freedom Evo automated pipetting machine controlled by EVOware™ Standard Software (Tecan Group AG, Männedorf, Switzerland) was used. To minimize any contamination, the tips were washed intensively with 7% v/v sodium hypochlorite (Carl Roth, Karlsruhe, Germany) in ultrapure water and ultrapure water before and after the pipetting steps. According to the manufacturer's instructions, 102 *μ*L of the cDNA mix were diluted in 1,248 *μ*L RNase free water and then placed in the Freedom Evo automated pipetting machine for adding 1,350 *μ*L of 2x RT^2^ SYBR® Green ROX qPCR Mastermix (Qiagen, Hilden, Germany). The mixture was mixed by gentle inversion to avoid bubbles. 25 *μ*L of this mix were pipetted in each well of the following 96-well RT^2^ Profiler™ PCR Arrays: Human-Induced Pluripotent Stem Cells, Human Neurogenesis, Human Neurotransmitter Receptors, Human Neutrophins and Receptors, Human Neuronal Ion Channels, and a custom-designed plate for analysis of genes involved in motoneurogenesis (Qiagen, Hilden, Germany). Each plate was sealed with Optical Thin-Wall 8-Cap Strips (Qiagen, Hilden, Germany). qRT-PCR analysis was performed with Eppendorf Mastercycler® epgradient S realplex^2^ with Mastercycler ep realplex software (Eppendorf AG, Hamburg, Germany) by activating polymerase for 10 min at 95°C, followed by 40 cycles of 15 s at 95°C and 1 min at 60°C. After each run, the threshold was adjusted to 200 and drift correction was enabled for better comparison. All experiments were repeated three times independently.

For data analysis, *C*_*t*_ values were exported to GeneGlobe (http://www.qiagen.com/geneglobe) using C_T_ cut-off of 35, RPLP0 housekeeping gene for normalization, fold regulation cut-off of 2.0, and *p* value cut-off of 0.05. The calculation of *p* value is based on an unpaired Student's *t*-test. The software RStudio (version 4.0.3 (2020-10-10)) was used for graphic presentation (RStudio Inc., Boston, USA).

## 3. Results

### 3.1. Identity of Generated hiPSC and Differentiated NPC

To verify successful hPDF nucleotransfection with pluripotency inducing factors, the GFP signal was detected one day after transfection (Supplementary Figure 1(a)). Over a cultivation period of 30 days in mTeSR™1, clearly defined hiPSC clones with distinct and compact morphology evolved from transfected single-cell hPDF (Supplementary Figure 1(b)). As shown in Supplementary Figure 2(a), hiPSC displayed alkaline phosphatase activity and were stained positive for pluripotency markers such as TRA-1-60, Oct3/4, and Nanog [15] (Supplementary Figures 2(b) and 2(c)). Furthermore, hiPSC were able to differentiate into cells morphologically similar to cells of the three germ layers (Supplementary Figure 1(c)). Genes coding for pluripotency, e.g., GDF3, FGF4, DNMT3B, TDGF1, and Nanog were significantly upregulated compared to hPDF (fold regulation ≥ 2.0; *p* < 0.05), which illustrates successful reprogramming to hiPSC (Supplementary Figure 3) [16].

**Figure 1:**
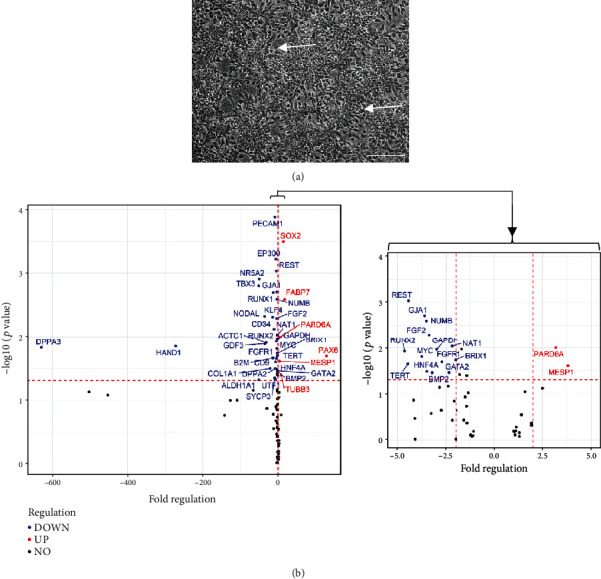
NPC differentiation from hiPSC for the generation of mature MNs. (a) NPC form neural rosettes illustrated in a representative phase-contrast image. Scale bar, 200 *μ*m. (b) Identity of NPC assessed by significant upregulation of genes involved in neurogenesis and downregulation of genes coding for pluripotency compared to hiPSC. Fold regulation of genes (≥2.0 or ≤ -2.0 in combination with *p* < 0.05) is shown as means (*n* = three independent experiments). The horizontal red dashed line indicates a *p* value of 0.05 whereas the vertical red dashed lines correspond to a fold regulation of -2.0 and 2.0, respectively. For better illustration, the range between -5.0 and 5.0 is additionally displayed.

After 21 days of inhibition of dual-SMAD signalling, NPC undergoing neuronal rosette formation (Figure 1(a)) were compared to their hiPSC-origin cells by gene expression analysis. Significant upregulation was found in genes involved in early motor neurogenesis, e.g., Pax6, FABP7, Sox2, and Tubb3 (fold regulation ≥ 2.0; *p* < 0.05) (Figure 1(b)) [17, 18]. In contrast, genes coding for pluripotency such as NODAL, DPPA3, and TBX3 were significantly downregulated (fold regulation ≤ −2.0 in combination with *p* < 0.05).

### 3.2. Morphological Pattern of the Two Differentiation Approaches

**Figure 2:**
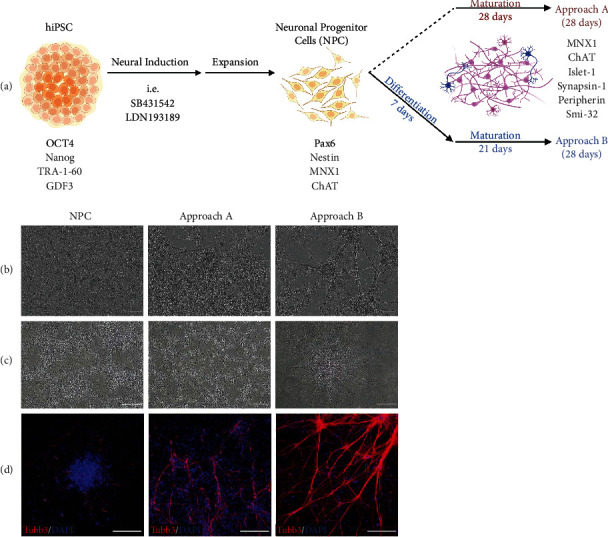
Morphological characteristics of neurons generated via approaches A and B compared to NPC. (a) A schematic overview of approaches A and B differentiating hiPSC-derived NPC into MNs, created with http://BioRender.com. (b) Representative images of cells of approach A and B captured with the IncuCyte® showing MN-type clusters evolving from homogeneous NPC culture. (c) Neuronal identity was confirmed for both approaches via Bielschowsky silver staining, illustrated by brown-stained nuclei and axons. (d) Immunofluorescent staining for Tubb3 (beta-3-tubulin; red) revealed pronounced neuronal network formation via both approaches. Cell nuclei were counterstained with DAPI. All scale bars, 200 *μ*m.

A scheme of 28 days NPC differentiation by direct maturation, approach A, and with a preceding differentiation step, approach B, is shown in Figure 2(a). Analyzed by IncuCyte®, both approaches yielded branched networks with MN-type clusters after 28 days of differentiation in comparison to NPC (Figure 2(b)). These clusters were confirmed as neuronal networks by Bielschowsky silver staining (Figure 2(c)) and immunostaining for the neuronal marker Tubb3 (Figure 2(d)). Approach B produced denser filaments than approach A, particularly evident in Tubb3 staining.

### 3.3. Variation in Neuronal Gene Expression Tested via qRT-PCR

**Figure 3:**
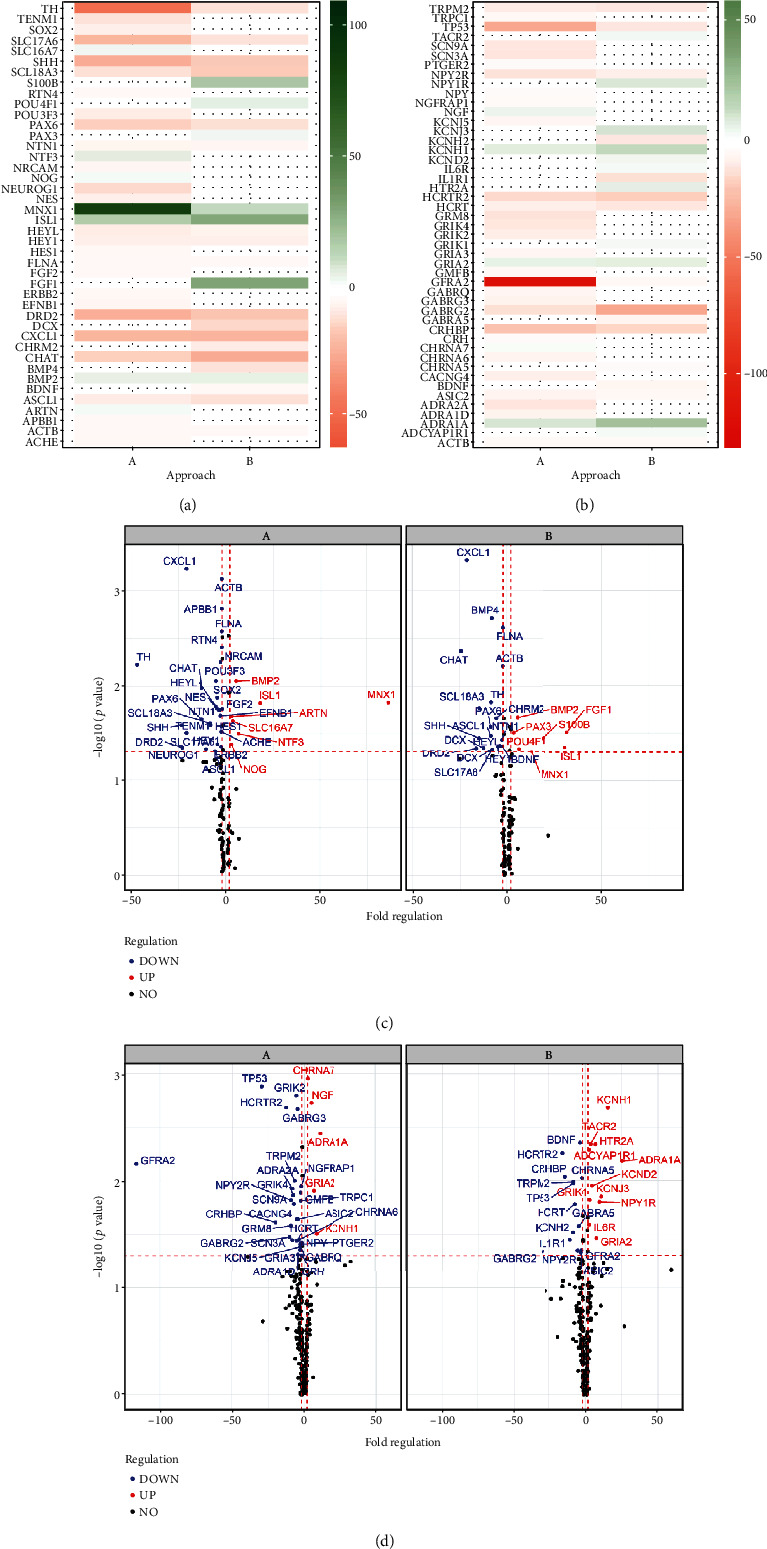
Neuronal gene expression pattern of cells of approach A and B. Genes involved in motor neurogenesis (a, c) and genes associated with the expression of neuronal ion channels, neurotransmitters, and neurotrophins (b, d) were differently expressed in approaches A and B compared to NPC, tested by qRT-PCR. Results are shown in a heat map (a, b), where red indicates significant downregulation while green represents significant upregulation of genes, and in a volcano plot (c, d), where a *p* value of 0.05 is indicated by a horizontal red dashed line and a fold regulation of -2.0 and 2.0 is represented by vertical red dashed lines (*n* = three independent experiments).

To further characterize the neurons derived from 28 days differentiation via approaches A and B, the expression of various genes characteristic of motor neurogenesis [19], neuronal ion channels [20], neurotrophins [21], and neurotransmitters [22] was determined. As shown in Figure 3, genes were significantly differentially expressed in cells of both approaches A and B compared to NPC (fold regulation ≥ 2.0 and ≤ -2.0 in combination with *p* < 0.05).

In approach A, some genes were selectively downregulated, including Sex Determining Region Y Box 2 (Sox2; -4.71), neurofilament protein Nestin (NES; -4.83), transcription factor Neurogenin 1 (NEUROG1; -10.62), and Hes Family BHLH Transcription Factor 1 (HES1; -2.43), which are associated with a neural progenitor fate [23, 24]. Genes coding for ion channels like Voltage-dependent Calcium Channel Gamma-4 Subunit (CACNG4; -5.38), Sodium Voltage-gated Channel Alpha Subunit 3 (SCN3A; -8.52), and Glutamate Ionotropic Receptor Kainate Type Subunit 2 (GRIK2; -5.83) and Subunit 4 (GRIK4; -7.82) were also found downregulated. Other genes involved in neurogenesis and neurotransmission, e.g., Noggin (NOG; 2.86), Neurotrophin 3 (NTF3; 6.53), Nerve Growth Factor (NGF; 4.75), Solute Carrier Family 16 Member 7 (SLC16A7; 3.45), and Cholinergic Receptor Nicotinic Alpha Subunit 7 (CHRNA7; 2.29) were found selectively upregulated.

In comparison, some genes involved in neurotransmission including Brain Derived Neurotrophic Factor (BDNF; -2.33), Cholinergic Receptor, Muscarinic 2 (CHRM2; -5.72), Gamma-Aminobutyric Acid Type A Receptor Subunit Alpha 5 (GABRA5; -4.61), Potassium Voltage-Gated Channel Subfamily H Member 2 (KCNH2; -8.13), and doublecortin (DCX; -9.46), a gene involved in neuronal migration, were selectively downregulated in approach B. Other genes associated with neurotransmission such as 5-Hydroxytryptamine Receptor 2A (HRT2A; 6.93), Potassium Inwardly Rectifying Channel Subfamily J, Member 3 (KCNJ3; 10.98), and Glutamate Ionotropic Receptor Kainate Type Subunit 1 (GRIK1; 2.99) were found selectively upregulated. Besides, an increased expression of genes encoding, e.g., Fibroblast Growth Factor 1 (FGF1; 31.60), POU Class 4 Homeobox 1 (POU4F1; 6.32), Paired Box Gene 3 (PAX3; 3.37), and S100 Calcium Binding Protein B (S100B; 19.27) was observed.

Concerning both approaches A and B, especially genes coding for motoneuronal proteins such as Motor Neuron And Pancreas Homeobox 1 (MNX1; A/86.42; B/12.95) and Insulin gene enhancer protein Islet-1 (ISL1; A/17.94; B/30.59) as well as genes coding for Glutamate Ionotropic Receptor AMPA Type Subunit 2 (GRIA2; A/6.48; B/7.62), Bone Morphogenetic Protein 2 (BMP2; A/5.20; B/5.63), Potassium Voltage-Gated Channel Subfamily H Member 1 (KCNH1; A/8.38; B/15.49), and Adrenoceptor Alpha 1A (ADRA1A; A/10.87; B/25.25) were upregulated.

Early differentiation marker Paired Box 6 (PAX6; A/-13.07; B/-8.72) was downregulated in both approaches as were genes associated with synaptic transmission such as Tyrosine Hydroxylase (TH; A/-46.90; B/-8.42), Choline O-Acetyltransferase (ChAT; A/-12.91; B/-24.36), Solute Carrier Family 18 Member A3 (SCL18A3/VAChT; A/-8.43; B/-14.69), Solute Carrier Family 17 Member 6 (SLC17A6/VGLUT2; A/-20.77; B/-7.84), and GDNF Family Receptor Alpha 2 (GFRA2; A/-116.43; B/-2.78). Furthermore, genes involved in neuronal differentiation and migration like Achaete-Scute Family, BHLH Transcription Factor 1 (ASCL1; A/-5.78; B/-8.59), Dopamine Receptor D2 (DRD2; A/-22.98; B/-16.40), and C-X-C Motif Chemokine Ligand 1 (CXCL1; A/-20.82; B/-21.23) as well as genes regulating Notch signalling such as Hes Related Family BHLH Transcription Factor With YRPW Motif Like (HEYL; A/-5.38; B/-4.00) and WNT signalling e.g., SHH (A/-23.39; B/-14.83) were less expressed in both approaches.

When comparing gene expression of cells differentiated with approach A for 28 days with cells differentiated with approach B for 28 days, only S100B (A/-8.99; *p* = 0.01), MNX1 (A/6.67; *p* = 0.03), and GFRA2 (A/-12.33; *p* < 0.05) were significantly differentially expressed in cells of approach A.

### 3.4. Expression of Early Differentiation Markers Pax6 and Nestin

**Figure 4:**
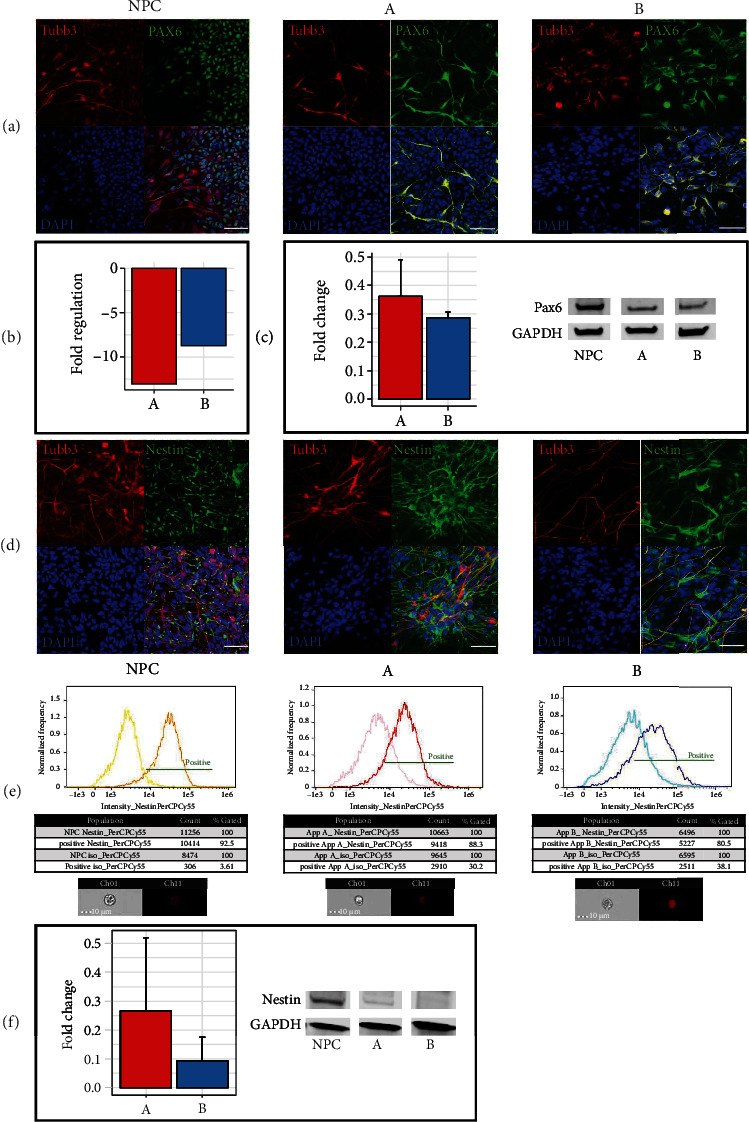
Early neuronal differentiation markers. (a) Representative confocal fluorescence microscopy images showing Pax6 (green) staining in the nuclei of NPC and Tubb3 (red) staining in the axons of all three approaches. Cell nuclei were counterstained with DAPI (blue). (b) Downregulation of Pax6 was detected by qRT-PCR in approaches A and B (*n* = three independent experiments). See Figure 3 for all results. (c) Decreased Pax6 protein expression was observed in Western Blot, shown in representative bands (*n* = three independent experiments). Each bar represents the fold change mean ± Standard Deviation (SD). (d) NPC and neurons of approach A and B show persistent Nestin (green) and Tubb3 (red) expression detected by immunofluorescence. Cell nuclei were counterstained with DAPI (blue). (e) Decreased Nestin expression was detected in approaches A and B by flow cytometry and (f) by Western Blot, shown in representative bands (*n* = three independent experiments). Each bar represents the fold change mean ± SD. All scale bars, 50 *μ*m.

To evaluate whether both approaches A and B were able to generate mature neurons after 28 days of differentiation, the expression levels of the early neuron differentiation markers Pax6 and Nestin were determined in comparison to NPC. As Nestin is described to be only expressed in uncommitted NPC and Pax6 is crucial for the generation and maintenance of NPC, both need to be downregulated to allow motoneuronal maturation [25, 26]. Pax6 was found downregulated, both at the mRNA level (A/-13.07; B/-8.72; *p* < 0.05) (Figure 4(b)) and the protein level (A/0.36 ± 0.13; B/0.29 ± 0.02) (Figure 4(c)). Nuclear staining for Pax6 was only observed in NPC but not in cells of approach A and B (Figure 4(a)), thus confirming the results of qRT-PCR and Western Blot.

Nestin expression was detected in NPC and cells of both approaches after 28 days of differentiation by immunofluorescence (Figure 4(d)). In flow cytometry, besides 92.5% of the NPC population, 88.3% of cells of approach A and 80.5% of approach B showed a Nestin-positive signal after 28 days of differentiation (Figure 4(e)). As approaches A and B were differentiated from NPC, the number of positively detected NPC was taken as the threshold for approaches A and B. This indicates Nestin downregulation in both approaches with detection of 58.1% positive cells in approach A and 42.4% positive cells in approach B compared to 92.5% positive NPC. Images of positive cells of each approach acquired during the flow stream are displayed. Detected by Western Blot, Nestin was downregulated in both approaches (A/0.27 ± 0.25; B/0.09 ± 0.08) after 28 days of differentiation (Figure 4(f)). These results confirm the quality of the generated NPC and suggest a successful differentiation of approaches A and B towards mature neurons.

### 3.5. Expression of the Motoneuronal Marker MNX1

**Figure 5:**
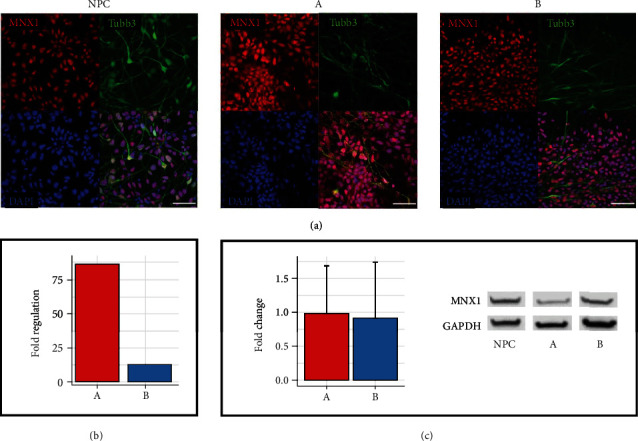
Motoneuronal differentiation marker MNX1. (a) Representative immunofluorescent images of MNX1 (red) staining show nuclear expression in NPC and neurons of approach A and B. Cell nuclei were counterstained with DAPI (blue). Scale bars, 50 *μ*m. (b) Detected via qRT-PCR, MNX1 was upregulated in both approaches in comparison to NPC (*n* = three independent experiments). See Figure 3 for all results. (c) Indifferent MNX1 protein expression was detected using Western Blot. Representative bands are shown from three independent experiments. Each bar represents the fold change mean ± SD.

To verify the generation of motoneuronal populations during 28-day differentiation of both approaches, the expression of MNX1, an early marker protein of postmitotic spinal MNs [27], was investigated. By immunofluorescence, MNX1 was detected in the cell nuclei of all three approaches (Figure 5(a)). These findings were confirmed by Western Blot results (Figure 5(c)) revealing no differences in protein expression between NPC and cells of both approaches (A/0.98 ± 0.70; B/0.92 ± 0.82). In contrast, qRT-PCR analysis revealed upregulation of MNX1 in cells of both approaches A and B in comparison to NPC (A/86.42; B/12.95; *p* < 0.05) (Figure 5(b)). Furthermore, MNX1 was significantly 6.67-fold higher expressed (*p* < 0.05) in cells of approach A compared to cells of approach B. These results suggest that motoneuronal precursor cells are also present in the early neuronal differentiation stage of NPC and indicate that SHH- and RA-signalling pathways have been activated during dual-SMAD inhibition.

### 3.6. Postmitotic Motoneuronal Markers ChAT and Islet-1

**Figure 6:**
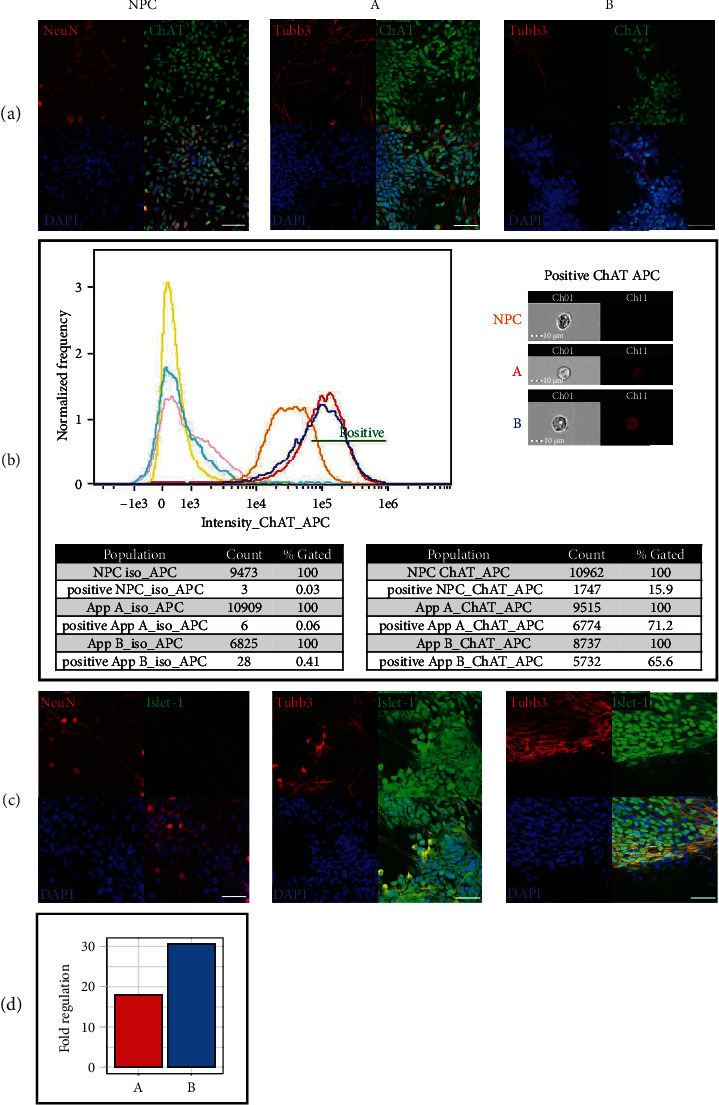
Late motoneuronal differentiation marker ChAT and Islet-1. (a) Immunofluorescent images show nuclear ChAT expression (green) in NPC and neurons of approach A and B. Cell nuclei were counterstained with DAPI (blue). Scale bars, 50 *μ*m. (b) In flow cytometry, cells of approach A and B revealed a higher ChAT-APC staining intensity compared to NPC. Representative images acquired during the flow stream highlight the difference in ChAT expression (*n* = three randomly selected images per group from three independent experiments). Scale bars, 10 *μ*m. (c) Islet-1 (green) is selectively expressed in neurons of approach A and B, as shown in representative immunofluorescent images. Cell nuclei were counterstained with DAPI (blue). Scale bar, 50 *μ*m. (d) Approach A and B show increased Islet-1 gene expression, detected by qRT-PCR (*n* = three independent experiments). See Figure 3 for all results.

To validate both differentiation approaches in terms of their efficiency to generate mature MNs, cells differentiated by approaches A and B for 28 days were tested for postmitotic motoneuronal markers ChAT and Islet-1 (ISL1) in comparison to NPC. By immunofluorescence and flow cytometry, all approaches were positively stained for ChAT (Figure 6(a)), an enzyme catalysing the synthesis of the neurotransmitter acetylcholine that is specifically found in cholinergic neurons as MNs of the spinal cord [3]. Images of cells of approach A and B, captured during flow stream, showed much higher ChAT-APC (Allophycocyanin) staining intensity in comparison to NPC, indicating that only cells of approach A and B should be considered positive. Thus, 71.5% of approach A and 65.6% of approach B were detected positive for ChAT-APC compared to 15.9% among NPC (Figure 6(b)).

ISL1, a transcription factor that is essential for the generation of mature and functional human MNs [3], was found in nuclei of cells of approach A and B but not in NPC detected by immunofluorescence (Figure 6(c)). In qRT-PCR, ISL1 was also significantly upregulated in both approaches (A/17.94; B/30.59; *p* < 0.05) in comparison to NPC, confirming the suitability of both approaches to form mature MNs (Figure 6(d)).

### 3.7. Neuronal Proteins Essential for Synaptic Transmission

**Figure 7:**
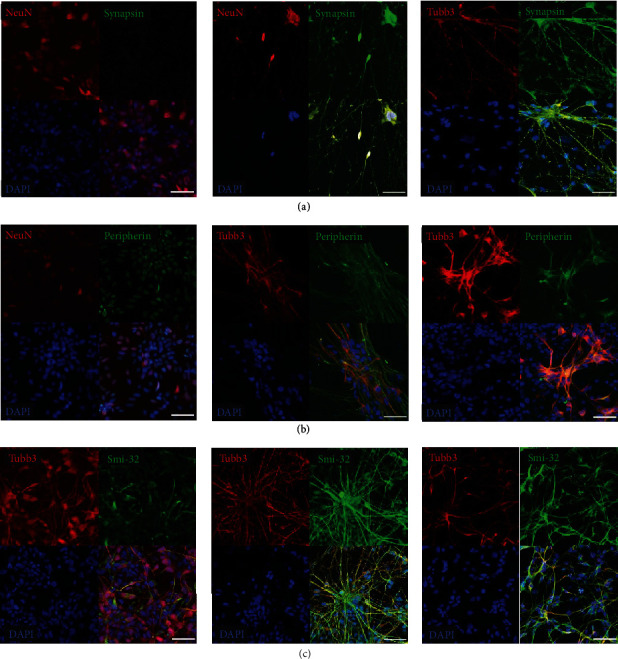
Expression of Synapsin-1, Peripherin, and Smi-32 indicate neuronal functionality. (a) Representative immunofluorescent images show Synapsin-1 positive synaptic vesicles stained in green puncta as well as (b) Peripherin- and (c) Smi-32-positive neuronal filaments (green) in neurons of approach A and B, which were not detected in NPC. Cell nuclei were counterstained with DAPI (blue) (*n* = three independent experiments). All scale bars, 50 *μ*m.

To test if both approaches A and B express proteins associated with mature MNs and neuronal functionality after 28 days of differentiation, the presence of Synapsin I (Syn I) Peripherin, and Neurofilament Heavy Polypeptide (Smi-32) was examined by immunofluorescence. Syn I belongs to the phosphoprotein family of synapsins, which play a key role in neurite outgrowth, synapse formation, and synaptic transmission [28] and is a characteristic marker for mature neurons [29]. Positive staining for Syn I in the form of puncta, which are presumed to be synaptic vesicles, was found in approach A and B, but not in NPC (Figure 7(a)), indicating maturity and assuming electrophysiological activity of differentiated cells of both approaches [30]. Peripherin expression was observed in neurons of the spinal cord [31] and is considered to be involved in neurite elongation during neuronal development [32]. Immunofluorescent results show Peripherin-positive neuronal filaments in approach A and B (Figure 7(b)). As a type III neurofilament protein, the function of Peripherin is affiliated with the postmitotic marker Smi-32 to form filament networks [33]. As illustrated in Figure 7(c), nuclei and axons of approach A and B were positively stained for Smi-32 indicating that functional networks were generated.

## 4. Discussion

This study gives insight into the potential of two differentiation approaches A and B using standardized, chemically defined, and ready-to-use culture media to generate mature MNs from foodprint- and integration-free derived hiPSC. Both differentiation approaches avoid the use of antibiotics or feeder cells, thus the derived MNs may have the potential to be implemented in the therapy of motor neurodegenerative diseases. Since hiPSC differentiation into MNs is a multiple-step process including the formation of NPC, the identification and characterization of these generated NPC is essential.

NPC were differentiated successfully under serum-free conditions from hiPSC, expressing early neuron differentiation markers such as Nestin, Sox2, Pax6, FABP7, and Tubb3 assessed by qRT-PCR, Western Blot, immunocytochemistry, and flow cytometry [34]. Nestin, a class VI intermediate filament protein and Sox2 are proteins characteristically occurring in NSC [35]. Expression levels of Pax6, FABP7, and Tubb3 are increased in the developing spinal cord and are early induced in differentiating MNs [18]. In particular, Pax6 plays a key role in patterning neuroectoderm cells into ventral spinal progenitors [36]. Interestingly, MNX1, a specific marker for pre- and postmitotic MNs [37] and described as being expressed in the developing spinal cord [38], was detected in NPC indicating that it was possible to generate NPC with a spinal fate after dual-SMAD inhibition. Jordan et al. [37] observed MNX1 expression after treatment with bFGF, which is well-known to induce proliferation of NSC and might be a supplement of the standardized NPC expansion medium used here. Surprisingly, ChAT was detected at a low level in NPC by flow cytometry and immunofluorescence. This also confirms the neural conversion towards MNs and might also be related to growth factors as Nistor et al. [39] found an increased number of ChAT-expressing NPC after treatment with bFGF and FGF8.

Maturation of MNs was achieved via both differentiation approaches A and B. Downregulation of Pax6 and Nestin is described in the course of MN maturation [25, 40] and was observed in both approaches, as shown in the results of qRT-PCR, Western Blot, immunofluorescence, and flow cytometry. Concerning MN morphology, a hallmark in the evaluation of MNs [41], approach A and B can be distinguished by their level of maturity. MN maturation is morphologically associated with extending long projections [3] and an increase in complexity in neurite outgrowth [42]. Although this was confirmed in both approaches by the IncuCyte® and neuron-specific staining for Tubb3, the approach A showed less dense neuronal filaments and less intense brown Bielschowsky staining compared to approach B which could be an indicator of a lower degree of maturation.

Concerning the expression profile, both approaches differ in aspects of neurotrophic proteins (e.g., GFRA2, BDNF, NTF3, and FGF1). These are involved in the regulation of neuronal survival, axon outgrowth, dendritic pruning, and synaptic plasticity [43, 44] and therefore are responsible for the development and maintenance of neuronal functionality. As GFRA2 is downregulated in postnatal spinal MNs [43], reduced GFRA2 expression indicates successful MN maturation. Furthermore, a reduced MN survival rate was found to be related to loss of BDNF, NTF3, and NTF4 [43]. As BDNF is downregulated in approach B and NTF3 is upregulated in approach A, this could indicate that MNs generated by approach A possess a higher level of robustness. Investigations on the NTF4 expression level would be needed for clarification. Concerning neurotrophin FGF1, Renaud et al. [44] described that increased expression levels can be found in MNs, which reach a maximum in adult neuronal tissues. As FGF1 is selectively upregulated in approach B, this may confirm approach B being more mature. Another protein involved in neuronal plasticity and long-term potentiation is S100b. It prevents developmental cell death in MNs [45] and was found upregulated in approach B. Those findings put the assumption that the approach A may be more robust into perspective.

Characteristic proteins essential for the identification of postmitotic and functional MNs are MNX1, ISL1, and ChAT [46]. MNX1 (also known as Hb9) promotes the specification of MNs and is considered to be a marker for lower MNs [47, 48]. Neurons obtained via approach A showed increased MNX1 mRNA level compared to approach B, suggesting that a higher amount of lower MNs was generated. Investigation on MNX1 protein level revealed no significant difference between both approaches, which relativises this assumption. In the further course of MN-specification, the expression of MNX1 determines the expression of the LIM homeodomain transcription factor ISL1 in postmitotic MNs [49]. ISL1 is one of the first motoneuronal genes expressed in postmitotic MNs and plays a key role in the specification of functional MNs by being involved in neurotransmitter expression and MN migration [3, 50]. ISL1 gene and protein expression was significantly increased in both approaches, confirming the generation of putative functional MNs. Furthermore, ISL1 regulates the expression of cholinergic genes such as VAChT, CHT, and ChAT [51]. ChAT is crucial for the synthesis of the neurotransmitter acetylcholine and is selectively expressed in MNs of the spinal cord [3]. Several studies found that ChAT-positive neurons possess electrophysiological activity [27, 46, 52–54]. On protein level, ChAT was detected in both approaches by flow cytometry and immunohistochemistry. In contrast, ChAT gene expression levels were decreased in both approaches, which may be due to posttranscriptional regulation in process of maturation [55]. These findings were also observed in rats by Corsetti et al. [53]. As ChAT is not expressed in the upper MNs, this may suggest successful NPC differentiation into mature and functional lower MNs [52, 56].

Genes coding for neuronal ion channels being upregulated in approaches A and B can be considered for the functional classification of mature neurons [57]. KCNH1, encoding for the voltage-gated potassium channel Kv10.1, is associated with neurotransmitter release and synaptic transmission in MNs [58, 59]. ADRA1A, coding for adrenergic receptor, and GRIA2, coding for glutamate ionotropic receptor, are involved in neurotransmitter release of catecholamines and glutamate and are essential for maintenance of functional MNs [60, 61]. Increased expression of KCNH1, ADRA1A, and GRIA2 in both approaches indicates successful maturation which is further underlined by decreased expression of genes coding for SLC17A6 and DRD2 [61, 62]. Further proteins associated with neural electrophysiological activity are Smi-32, Peripherin, and Syn I. Expression of Smi-32 is specific for MNs of the spinal cord and was found in cytoplasm and dendrites of both approaches [3]. Structural support and transport of nutrients are the main functions of neurofilament Smi-32. Smi-32 and Peripherin, a type III intermediate filament protein appearing during MN development, are functionally interdependent and assure normal conduction velocity [31, 33]. Thus, both proteins are essential for the formation of functional MNs. The detection of scattered synaptic puncta on dendrites is characteristic for immunostaining of Syn I, a synaptic vesicle protein implicated in synapse formation and synaptic transmission [28, 63]. Syn I is selectively expressed in mature neurons and was found to increase the amplitude of evoked synaptic currents [28]. Thus, the detection of Smi-32, Peripherin, and Syn I confirms maturation and suggests functional activity of both approaches [33, 64, 65].

To clarify if the generated MNs possess functionality, not only investigations on electrophysiological activity using, e.g., patch-clamp or multielectrode-array system need to be performed but also coculture experiments with skeletal muscle cells are required as the proper synaptic and signalling context is provided by neuromuscular junctions [27]. Considering therapy of motoneuronal degeneration diseases, Trawczynski et al. [2] suggest that particularly promising results are achieved by transplanting MN precursor cells which form appropriate synapses and exhibit better functional recovery compared to postmitotic MNs. By using xeno-free matrices, the method presented here for generating MN precursor cells constitutes a promising tool for regenerative medicine. In the future, it will be interesting to sort differentiated MNX1-positive motoneuronal precursor cells and to investigate their ability to differentiate into mature and functional MNs *in vivo*.

## 5. Conclusion

This study demonstrated that MNX1- and ChAT-positive MN precursors can be differentiated from integration-, feeder-, serum-, and antibiotic-free generated hiPSC by dual-SMAD inhibition and subsequent expansion. The derived MN precursor cells form mature and putative functional MNs after application of chemically defined, standardized, and antibiotic-free culture media. The quality of hiPSC, MN precursors, and mature MNs was continuously confirmed in terms of their morphology, protein, and gene expression, ensuring a robust and reproducible protocol. Comparison of differentiation approaches A and B revealed a significant difference in MNX1 gene expression. Thus, the approach A appears to be a more efficient differentiation method that might set the course for clinical translation if the entire process of hiPSC generation and differentiation, including all culture media and matrices used, complies with cGMP requirements.

## Data Availability

Data may be available from the corresponding author upon reasonable request.

## Supplementary Materials

Supplementary Materials Supplementary Figure 1: generation of hiPSC from hPDF. Supplementary Figure 2: expression of pluripotency associated markers. Supplementary Figure 3: gene expression of hiPSC derived from hPDF. Supplementary Method 1: expansion of pluripotency inducing plasmids. Supplementary Method 2: qualification of plasmids for hiPSC generation. Supplementary Method 3: alkaline phosphatase live staining for hiPSC identification. Supplementary Method 4: cryosection of hiPSC for immunofluorescence of TRA-1-60 and Oct 3/4. Supplementary Method 5: uDISCO method for immunofluorescence of Nanog in hiPSC. Supplementary Method 6: RNA extraction from hiPSC.
