# Supplementary material for: The Efficiency of Direct Maturation: the Comparison of Two hiPSC Differentiation Approaches into Motor Neurons
Source: Stem Cells Int. 2022 Dec 9;2022:1320950. doi: 10.1155/2022/1320950 (PMC9757946; doi:10.1155/2022/1320950)
Supplement: Supplementary Materials — Supplementary Figure 1: generation of hiPSC from hPDF. Supplementary Figure 2: expression of pluripotency associated markers. Supplementary Figure 3: gene expression of hiPSC derived from hPDF. Supplementary Method 1: expansion of pluripotency inducing plasmids. Supplementary Method 2: qualification of plasmids for hiPSC generation. Supplementary Method 3: alkaline phosphatase live staining for hiPSC identification. Supplementary Method 4: cryosection of hiPSC for immunofluorescence of TRA-1-60 and Oct 3/4. Supplementary Method 5: uDISCO method for immunofluorescence of Nanog in hiPSC. Supplementary Method 6: RNA extraction from hiPSC. [file 1320950.f1.docx]

**Supplementary Materials**

**The efficiency of direct maturation - The comparison of two hiPSC differentiation approaches into motor neurons**

Catherine Schaefers^1^, Simone Rothmiller^1^, Horst Thiermann^1^, Theo Rein^2^, Annette Schmidt^1,3^

¹Bundeswehr Institute of Pharmacology and Toxicology, Neuherbergstr. 11, 80937 Munich, Germany.

²Max Planck Institute of Psychiatry, Kraepelinstr. 2‑10, 80804 Munich, Germany.

³Institute of Sport Science, University of the Bundeswehr Munich, Werner‑Heisenberg‑Weg 39, 85577 Neubiberg, Germany.

Correspondence should be addressed to Annette Schmidt, annette.schmidt@unibw.de

**List of Content**

**Supplementary Figures**

**Supplementary Figure 1: Generation of hiPSC from hPDF.**

**Supplementary Figure 2: Expression of pluripotency associated markers.**

**Supplementary Figure 3: Gene expression of hiPSC derived from hPDF.**

**Supplementary Materials and Methods**

**Supplementary Method 1: Expansion of pluripotency inducing plasmids.**

**Supplementary Method 2: Qualification of plasmids for hiPSC generation.**

**Supplementary Method 3: Alkaline phosphatase live staining for hiPSC identification.**

**Supplementary Method 4: Cryosection of hiPSC for immunofluorescence of TRA-1-60 and Oct 3/4.**

**Supplementary Method 5: uDISCO method for immunofluorescence of Nanog in hiPSC.**

**Supplementary Method 6: RNA extraction from hiPSC.**


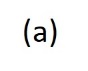
**Supplementary Figures**


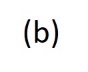

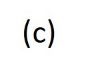

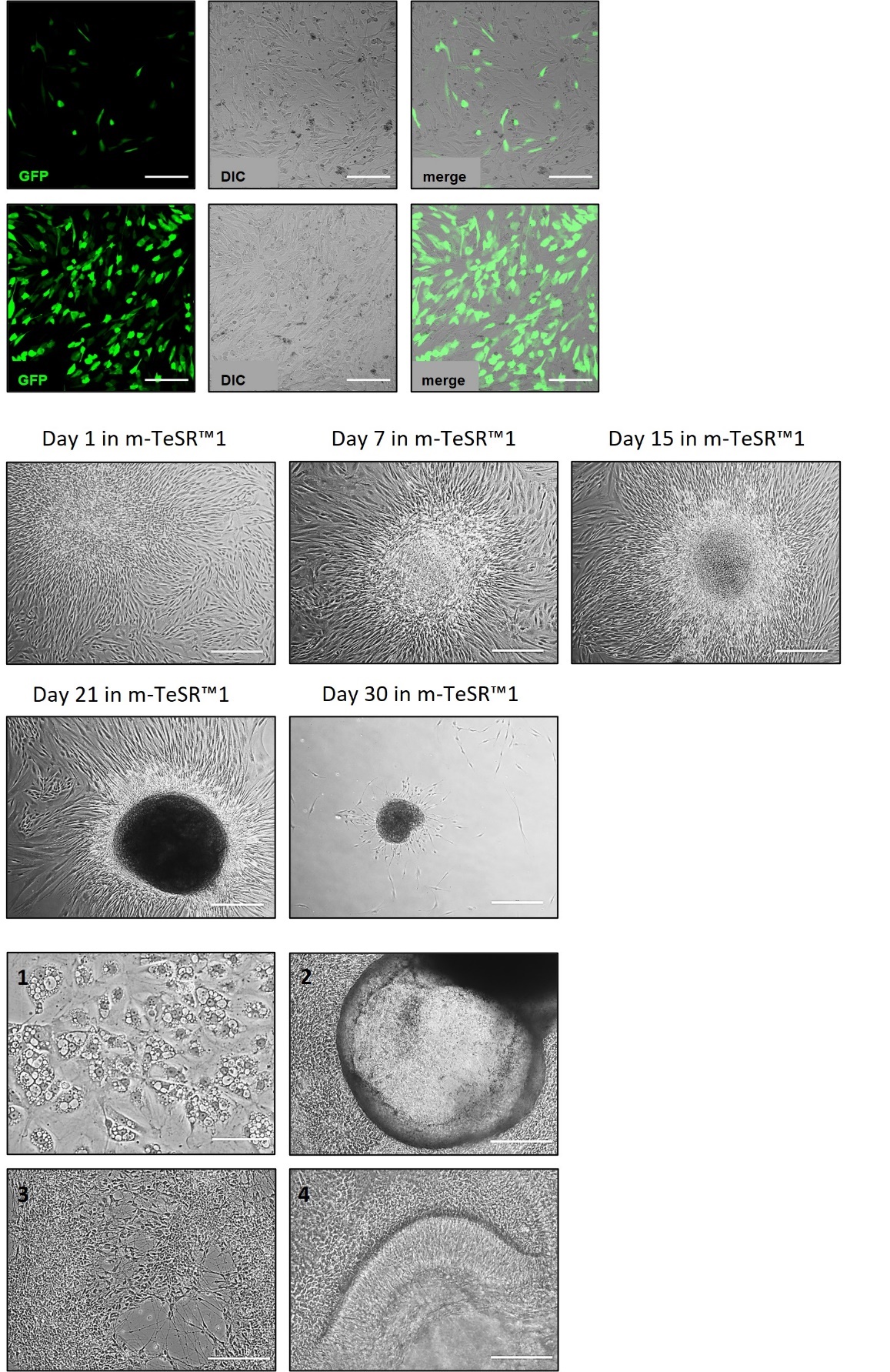


**Supplementary Figure 1: Generation of hiPSC from hPDF.**

(a) Representative immunofluorescence images of GFP Signal (green) from successfully electroporated hPDF with three plasmids inducing pluripotency including GFP plasmid (top) and GFP plasmid only (bottom) captured one day after transfection. Scale bar, 200 µm. (b) Representative phase‑contrast images showing the evolution of distinct hiPSC clones over a period of 30 days in mTeSR™1. Scale bar, 500 µm. (c) Representative images morphologically illustrate the ability of the generated hiPSC to differentiate into cells resembling the cells of the three germ layers. Mesoderm indicated by adipocyte‑like cells (1), endoderm implied by bladder structure without epithelial layer (2), ectoderm represented by neuronal cells (3) and epithelial‑like structures built from cells of all three germ layers (4). Scale bars, 200 µm (1) and 500 µm (2, 3, 4).


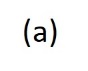

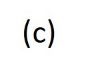

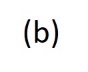

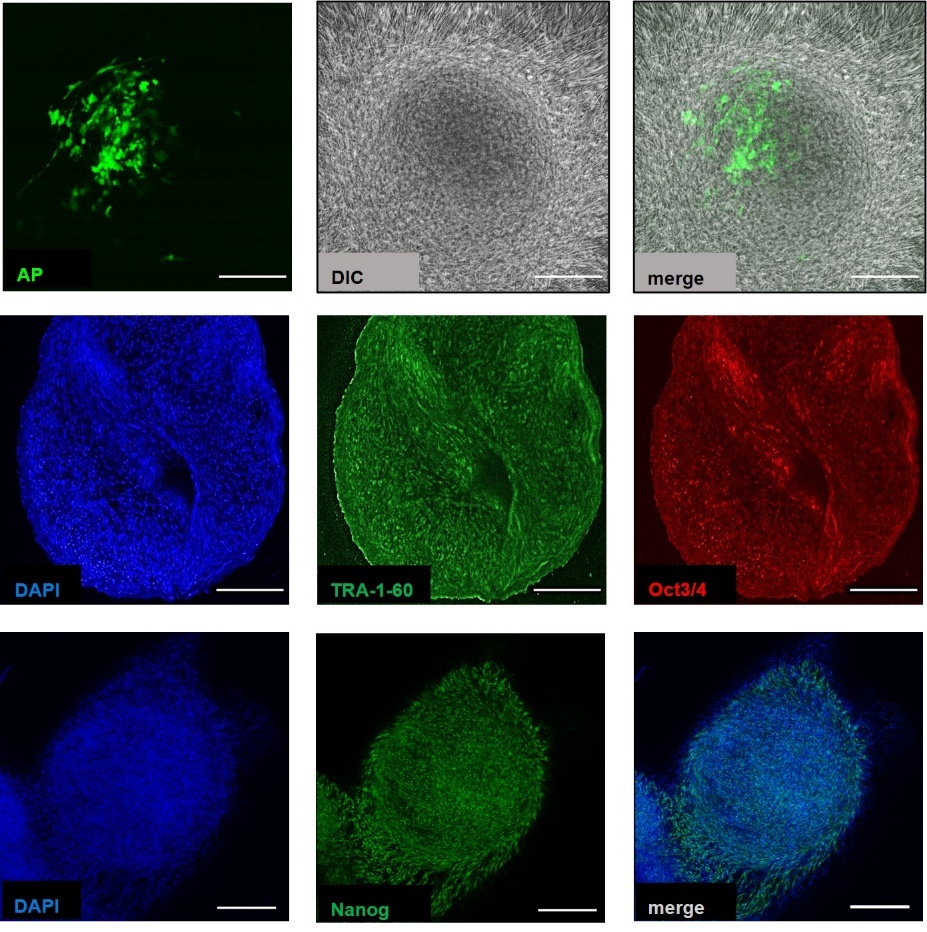


**Supplementary Figure 2: Expression of pluripotency‑associated markers.**

(a) Representative immunofluorescent images show Alkaline Phosphatase activity (AP; green) in hiPSC 20 days after transfection. (b) Immunofluorescent detection of TRA‑1‑60 (green), Oct 3/4 (red), as well as (c) Nanog (green) in cell nuclei indicates successful reprogramming to hiPSC illustrated in representative images. Cell nuclei were counterstained with DAPI. All scale bars, 200 µm.


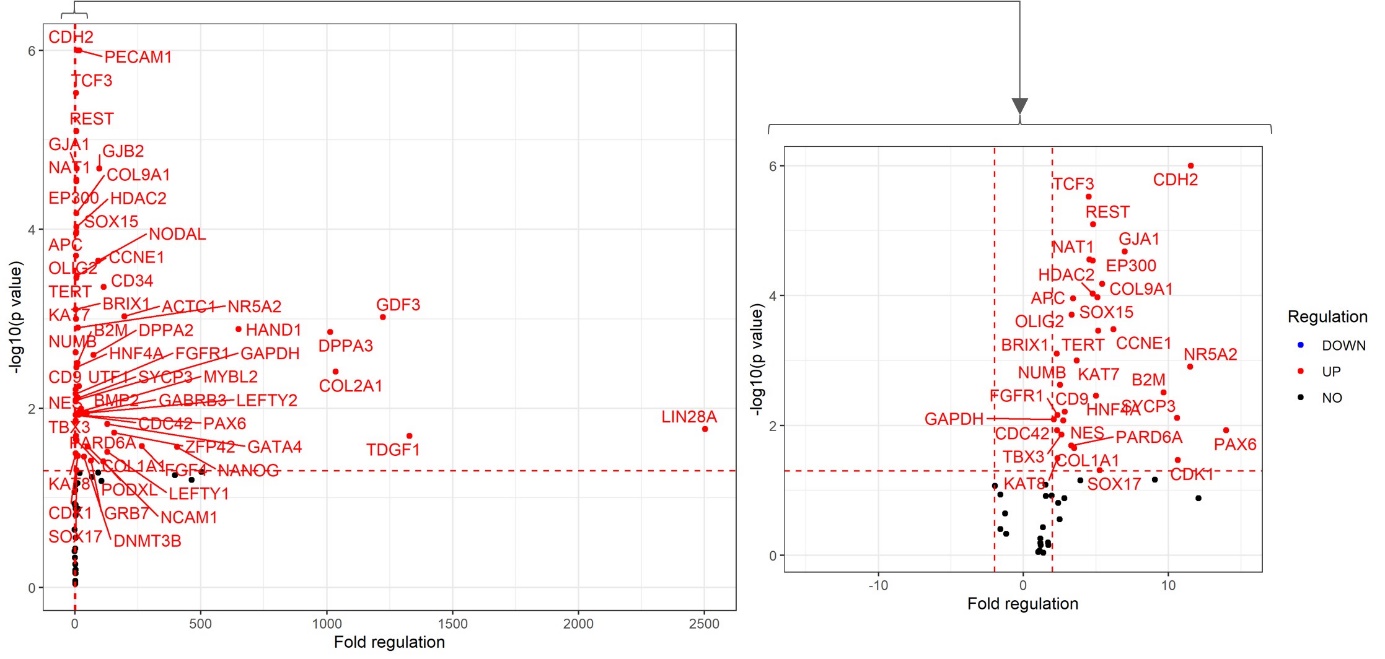
**Supplementary Figure 3: Gene expression of hiPSC derived from hPDF.**

Genes associated with pluripotency were upregulated in hiPSC after 30 days of cultivation in mTeSR™1 compared to hPDF. After mRNA extraction from hiPSC, a qRT‑PCR assay was performed. Fold regulation of genes (≥ 2.0; p < 0.05) is shown as means (n = three independent experiments). The horizontal red dashed line indicates a p value of 0.05 whereas the vertical red dashed lines correspond to a fold regulation of ‑2.0 and 2.0, respectively. For better illustration, the range between ‑10.0 and 10.0 is additionally displayed.

**Supplementary Materials and Methods**

**Supplementary Method 1: Expansion of pluripotency inducing plasmids.**

Four DH5α-E.coli strains, each containing one of the following plasmids as described by Okita et al. [65] pCXLE-hOCT3/4‑shp53-F, pCXLE-hSK, pCXLE-hUL and pCXLE-EGFP (Addgene, Watertown, Massachusetts, USA), were thawed on ice. 10 µL of each strain was added separately to 40 mL LB-Amp-medium (LB-Medium according to Miller [AppliChem, Darmstadt, Germany] with 100 µg/mL Calbiochem® sterile ampicillin solution [100 mg/mL, Merck, Darmstadt, Germany]) and incubated overnight at 37 °C and 220 rpm. Using an inoculation loop, the bacterial suspensions were plated on LB-Amp-agar plates (LB‑Agar according to Miller [AppliChem, Darmstadt, Germany] with 100 µg/mL Calbiochem® sterile ampicillin solution [100 mg/mL, Merck, Darmstadt, Germany]) and incubated overnight at 37 °C. Subsequently, five colonies of each strain were picked and expanded by incubation overnight at 37 °C and 220 rpm in 10 mL LB‑Amp-medium. Bacterial cells were obtained by centrifugation at 6,000 x g for 10 min at 4 °C. Isolation of plasmids was performed with QIAprep Spin Miniprep Kit (Qiagen, Hilden, Germany) according to manufacturer’s protocol. Briefly, the cell pellets were resuspended in 250 µL buffer P1 and mixed with 250 µL buffer P2. 350 µL buffer P3 was added and carefully mixed by inverting. Cell debris was separated by centrifugation for 10 min at 17,900 x g. 800 µL of the supernatant was transferred to a QIAprep Spin Column. The column was washed with 0.75 mL buffer PE and was dried by centrifugation at 21,135 x g. Bound DNA was eluted by adding 50 µL of nuclease free water and centrifugation at 21,135 x g. DNA concentration was determined using NanoQuant Plate™ with Plate Reader infinite M200 Pro (Tecan Group AG, Männedorf, Switzerland).

**Supplementary Method 2: Qualification of plasmids for hiPSC generation.**

500 ng of each pluripotency‑inducing plasmid was mixed separately with 2 µL of 10 x buffer EcoRI, 1 µL EcoRI (10 U/µL), and nuclease free water to a total volume of 20 µL in a PCR clean 0.2 mL tube and incubated for 2 h at 37 °C. Enzyme EcoRI was inactivated by incubation for 20 min at 65 °C. DNA fragments were separated on a 1.5 % GelPilot® LE Agarose Gel (Qiagen, Hilden, Germany) containing 0.3 µg/mL ethidium bromide (Sigma-Aldrich, St. Louis, Missouri, USA) with TAE buffer (Carl Roth, Karlsruhe, Germany) for 60 min at 140 V. The following DNA bands were to be expected: PP‑1 (OCT4; 1108 bp, 3346 bp, 6834 bp), PP‑2 (SOX2, KLF4; 2513 bp, 10180 bp), PP‑3 (L‑MYC; 1871 bp, 10180 bp), PP‑4 (EGFP; 732 bp, 10180 bp) and were detected under UV light.

**Supplementary Method 3: Alkaline phosphatase live staining for hiPSC identification.**

To facilitate the distinction and separation of hiPSC from hPDF, the Alkaline Phosphatase Live Staining (Invitrogen by Thermo Fisher, Waltham, Massachusetts, USA) was used according to the manufacturer’s instructions. In brief, hiPSC cultivated in mTeSR™1 (Stemcell Technologies, Vancouver, Canada) for 10 days and were washed twice in prewarmed DMEM/F‑12 for 2 min. Cells were stained by incubation for 25 min with 1x AP Live Stain working solution and subsequently washed twice with DMEM/F‑12 for 5 min. Positive colonies were visualized with ZEISS LSM 710 confocal microscope (ZEISS, Oberkochen, Germany).

**Supplementary Method 4: Cryosection of hiPSC for immunofluorescence of TRA-1-60 and Oct 3/4.**

After washing twice with PBS, hiPSC clones were manually picked each with a separate, sterile pipette tip and transferred to a Biopur® 1.5 mL tube each (Eppendorf, Hamburg, Germany) filled with 100 µL Killik OCT (Bio-Optica, Milan, Italy). Another 200 µL Killik OCT were added, and the mixture was frozen for 1 day at ‑20 °C. 10 µm slices were obtained using the Leica CM 1950 cryostat (Leica Biosystems, Wetzlar, Germany) at ‑15 °C. Excess cryostat medium was removed by washing the slices for 5 min in ultrapure water. Fixation, staining and analysis were performed as described previously (in “Immunofluorescence”). The following primary and secondary antibodies were used: Mouse Anti‑TRA‑1‑60 (#60064 Stemcell Technologies, Vancouver, Canada) 1:100, Rat Anti‑OCT3/4 (14‑5841‑82, Invitrogen by Thermo Fisher Scientific, Waltham, Massachusetts, USA) 1:100, Goat Anti‑Mouse IgG Antibody (H+L) Dylight® 594 (Vector Laboratories, Burlingame, California, USA) 1:800 and Sheep Anti‑Rat FITC (GTX26848, Genetex, Irvine, California, USA) 1:1,000. DAPI (422801, Biolegend, San Diego, California, USA) 1:75,000 was used for nuclear cell staining.

**Supplementary Method 5: uDISCO method for immunofluorescence of Nanog in hiPSC.**

hiPSC clones were cleared and stained using the method of ultimate 3D imaging of solvent-cleared organs (uDISCO) according to the protocol of Zaeck et al.[66]. All the following steps were conducted under gentle shaking and materials were purchased from Sigma-Aldrich, St. Louis, Missouri, USA. In brief, cells were washed twice with PBS for 30 min after fixation with 4 % w/v PFA in PBS for 1 h. iPSC clones were pre-treated with ascending concentrations of methanol (20 %,40 %, 60 %, 80 % methanol v/v in ultra‑pure water for 30 min each). After two times incubation with 100 % methanol for 30 min, cells were incubated for 10 min at 4 °C. Subsequently, cells were exposed to descending concentrations of methanol (80 %, 60 %, 40 %, 20 % v/v methanol in ultra‑pure water) for 30 min each, followed by washing with PBS and twice with PTx.2 solution (0.2 % v/v Triton X-100 in PBS) for 30 min each. Cells were incubated with permeabilization buffer (0.2 % v/v Triton X-100, 20 % v/v DMSO, 0.3 M glycine in PBS) for 1 day at 37 °C followed by blocking with blocking buffer (0.2 % v/v Triton X-100, 10 % v/v DMSO, 6 % (v/v) bovines serum albumin (BSA) in PBS) for 1 day at 37 °C. The following primary and secondary antibodies were used: Rabbit Anti‑Nanog (ab21624, Cambridge, UK) 1:1,000 and Goat Anti‑Rabbit IgG Antibody (H+L) Dylight® 488 (Vector Laboratories, Burlingame, California, USA) 1:800. For 5 days at 37 °C, cells were incubated with the primary antibody in antibody diluent with DMSO (3 % v/v BSA, 5 % DMSO in PTwH (0.2 % v/v Tween-20, 0.1 % heparin solution (10 mg/mL in ultra‑pure water) in PBS) with solution exchange after 2.5 days. All the following steps were carried out by exclusion of light. After washing with PTwH for 5 times until the next day, the secondary antibody was diluted in antibody diluent without DMSO and applied for 3 days at 37 °C. Cells were washed 5 times with PTwH until the following day. Nuclear staining was performed with DAPI (422801, Biolegend, San Diego, California, USA) 1:75,000 in PTwH for 5 h at RT, followed by washing with PTwH for 5 times until the next day. hiPSC were cleared by exposure to BABB-D15 (0.4 % v/v tocopherol in Diphenylether-BABB (1:15) with BABB (50 % v/v Benzyl alcohol, 50 % v/v Benzyl benzoate)) for 4 h at RT. Cells were analysed using ZEISS LSM 710 confocal microscope (ZEISS, Oberkochen, Germany).

**Supplementary Method 6: RNA extraction from hiPSC.**

Disruption and homogenization of five hiPSC clones was performed with 300 µl QIAzol® Lysis Reagent in a grinding tube (Sample Grinding Kit, Cytiva, Marlborough, Massachusetts, USA) for 3 min. Cell debris was removed by centrifugation for 3 min at 12,000 x g. According to the manufacturer’s instructions concerning QIAzol® Reagent, the supernatant was transferred to a Biopur® 1.0 mL tube (Eppendorf, Hamburg, Germany) and was incubated for 5 min. 60 µL chloroform (Sigma-Aldrich, St. Louis, Missouri, USA) was added to the mixture, mixed for 15 s, and incubated for 2 min on the benchtop. For phase separation, the tube was centrifugated for 15 min at 12,000 x g at 4 °C. The upper aqueous fraction was preserved in a new Biopur® 1.5 mL tube, mixed with 150 µL isopropanol (Sigma-Aldrich, St. Louis, Missouri, USA) and incubated for 10 min. RNA was pelleted by centrifugation for 10 min 12,000 x g at 4 °C. The pellet was resuspended in 500 µL 75 % ethanol (Sigma-Aldrich, St. Louis, Missouri, USA) and centrifuged for 5 min at 7,500 x g at 4 °C. After discarding the supernatant, RNA was dissolved in 30 µL RNase free water. Determination of RNA concentration, cDNA synthesis and qRT‑PCR analysis was performed as described before (see “Analysis of gene expression of Approach A and B in comparison to NPC”).
